# Using technology to reduce critical deterioration (the DETECT study): a cost analysis of care costs at a tertiary children's hospital in the United Kingdom

**DOI:** 10.1186/s12913-023-09739-3

**Published:** 2023-07-04

**Authors:** Eduardo Costa, Céu Mateus, Bernie Carter, Holly Saron, Chin-Kien Eyton-Chong, Fulya Mehta, Steven Lane, Sarah Siner, Jason Dean, Michael Barnes, Chris McNally, Caroline Lambert, Bruce Hollingsworth, Enitan D. Carrol, Gerri Sefton

**Affiliations:** 1 Nova School of Business and Economics, Carcavelos, Portugal; 2 Present Address: Lancaster University, Lancaster, UK; 3 Faculty of Health, Social Care and Medicine, Edge Hill University, Ormskirk, UK; 4 Alder Hey Children’s NHS Foundation Trust, Liverpool, UK; 5 University of Liverpool, Liverpool, UK; 6 Clinical Research Division, Alder Hey Children’s NHS Foundation Trust, Liverpool, UK; 7 Finance Department, Alder Hey Children’s NHS Foundation Trust, Liverpool, UK; 8 Institute of Infection, Veterinary and Ecological Sciences, University of Liverpool, Liverpool, UK; 9 Department of Infectious Diseases, Alder Hey Children’s NHS Foundation Trust, Liverpool, UK; 10 Intensive Care Unit, Alder Hey Children’s NHS Foundation Trust, Liverpool, UK

**Keywords:** Children’s critical care, Cost analysis, Critical deterioration events, Paediatric, Paediatric early warning system score, Paediatric early warning system

## Abstract

**Background:**

Electronic early warning systems have been used in adults for many years to prevent critical deterioration events (CDEs). However, implementation of similar technologies for monitoring children across the entire hospital poses additional challenges. While the concept of such technologies is promising, their cost-effectiveness is not established for use in children. In this study we investigate the potential for direct cost savings arising from the implementation of the DETECT surveillance system.

**Methods:**

Data were collected at a tertiary children’s hospital in the United Kingdom. We rely on the comparison between patients in the baseline period (March 2018 to February 2019) and patients in the post-intervention period (March 2020 to July 2021). These provided a matched cohort of 19,562 hospital admissions for each group. From these admissions, 324 and 286 CDEs were observed in the baseline and post-intervention period, respectively. Hospital reported costs and Health Related Group (HRG) National Costs were used to estimate overall expenditure associated with CDEs for both groups of patients.

**Results:**

Comparing post-intervention with baseline data we found a reduction in the total number of critical care days, driven by an overall reduction in the number of CDEs, however without statistical significance. Using hospital reported costs adjusted for the Covid-19 impact, we estimate a non-significant reduction of total expenditure from £16.0 million to £14.3 million (corresponding to £1.7 million of savings – 11%). Additionally, using HRG average costs, we estimated a non-significant reduction of total expenditure from £8.2 million to £ 7.2 million (corresponding to £1.1 million of savings – 13%).

**Discussion and conclusion:**

Unplanned critical care admissions for children not only impose a substantial burden on patients and families but are also costly for hospitals. Interventions aimed at reducing emergency critical care admissions can be crucial to contribute to the reduction of these episodes’ costs. Even though cost reductions were identified in our sample, our results do not support the hypothesis that reducing CDEs using technology leads to a significant reduction on hospital costs.

**Trial registration:**

Current Controlled Trials ISRCTN61279068, date of registration 07/06/2019, retrospectively registered.

**Supplementary Information:**

The online version contains supplementary material available at 10.1186/s12913-023-09739-3.

## Background

Paediatric intensive care admissions impose a substantial burden on children, carers and in the health system. These admissions are very costly, implying increasing financial strain to hospitals [1]. Moreover, the length of stay is usually identified as the key determinant for paediatric intensive care costs [2]. Hence, interventions aiming at reducing the length of stay or avoiding admissions, are likely to contribute to improved efficiency and cost savings.

Electronic early warning systems (e-EWS) have been used in adult health settings for many years to prevent critical deterioration events (CDEs). These systems are typically based on algorithms which analyse regular and observable data to predict patient deterioration [3, 4]. The use of technology to improve the accuracy, reliability and availability of patients’ vital signs is often associated with reduced mortality [5].

However, similar technologies have not yet been widely diffused for children in hospitals [6]. Nonetheless, outcomes for critical care paediatric patients change considerably depending on admission characteristics [7, 8]. The use of Paediatric Early Warning (PEW) scores and systems, which exploit this variability in admission characteristics and outcomes, has increased over the last decades. Still, its implementation has been inconsistent and evidence of effectiveness of PEWS have been hampered by paper-based implementation [9].

Some studies suggest that these types of systems can reverse an increasing trend of critical deterioration [10] and improve clinical outcomes [11]. However, a large international study, comparing paper-based bedside PEWS with usual care, did not find a significant decrease in mortality among hospitalised paediatric patients [12].

While the concept of e-EWS is promising, its cost-effectiveness is not established for use in children and the impact of these programs on hospital costs or profits is unclear [13, 14]. Most studies typically fail to provide detailed cost information, precluding a detailed analysis on the cost-effectiveness of these technologies.

This paper presents the cost-effectiveness findings from the DETECT study (Dynamic Electronic Tracking and Escalation to reduce Critical care Transfers) [6]. The DETECT study implemented a proactive end-to-end deterioration solution (the DETECT surveillance system) across a tertiary children’s hospital in the United Kingdom (UK). The DETECT surveillance system aims to proactively screen paediatric patients for early signs of serious deterioration or sepsis, thereby reducing complications and emergency transfers to critical care following deterioration in hospital. This paper provides evidence on the direct cost savings arising from the implementation of the DETECT surveillance system, which contributes to reduce CDEs.

## Methods

This paper aimed to explore cost savings with the implementation of the DETECT surveillance system. To estimate such costs, we relied on the comparison between patients in the baseline period (March 2018 to February 2019) and patients exposed to the DETECT surveillance system (March 2020 to July 2021). In each period, a total of 19,562 hospital admissions (excluding day-cases) were recorded.

The analysis was based on the quantification of costs associated with the CDEs for those hospital admissions. In the baseline period, a total of 324 CDEs were recorded in a set of 19,562 hospital admissions. In the post-intervention period, for the same overall number of hospital admissions (matched cohort), a total of 286 CDEs were identified within 225 different patients. In this paper, we quantified the direct hospital costs associated with these CDEs for the baseline period and compared them with the costs observed in the post-intervention period.

The analysis is performed with an hospital perspective, i.e., not accounting for costs outside the hospital setting. This implies that all direct costs recorded during each hospital admission were included in the analysis. We estimated changes in costs arising from the utilization of the DETECT surveillance system. These were estimated by comparing the costs for CDEs in the baseline and post-intervention period. Two data sources were used to value the resources consumed: a) The primary data source was based on hospital reported costs; b) National average costs, based on Healthcare Resource Group (HRG), were also used as a sensitivity analysis.

### Hospital reported costs

To understand the determinants of the costs associated with the CDEs, patient-level costs were reported by the hospital. These costs were generated by the cost accounting department at the hospital and included all direct health care costs allocated to each individual episode. To avoid bias from long hospital admissions, and according to the predefined protocol, costs per day for critical care and hospital stay following a CDE were capped at up to 90 days. All costs were reported in British pounds (GBP).

Hospital reported costs were generated by Alder Hey Children’s Hospital’s patient level information and costing system (PLICS) which has a “full assurance” audit rating from auditors EY – Ernst & Young and NHS Improvement. The system takes patient level information feeds for most departments in the Trust, which show what happened to each patient each day. Hospital reported costs were computed based on the NHS Costing Standards^1^, which are used by every NHS provider trust in England.

For example, ICU costs were allocated on a daily basis to everyone who was on ICU during the year on the basis of minutes they spent on the ward that day, weighted by the acuity of the patient that day. Similarly, biochemistry costs were allocated to all patients who had biochemistry tests, with each test weighted according to time spent and consumable cost. Therefore, the system can report the actual costs of the actual patients on the actual days that they were in critical care. This enabled us to aggregate the cost of each relevant patient on the days when they were in critical care.

### Health resource groups costs

Patient-level costs reported by the hospital may differ from national average costs. An important part of health economics analysis concerns whether those costs are generalizable to other hospitals in the National Health Service (NHS). Hence, we used national average costs, based on Healthcare Resource Groups (HRG), to compare patients in the baseline and in the post-intervention period. This improved the robustness of the analysis and the external validity of the results in the NHS.

Within the NHS, patient events which consume a similar level of resources are grouped in a HRG. These groups are used to compare activity across hospitals and have unit costs associated which influence reimbursement schemes. However, relative to hospital reported costs, national average costs are less precise since they represent the average cost of all critical care patients, rather than just the patients who had unplanned admissions to critical care.

National average costs for hospital episodes are published under the National Cost Collection. This collection includes aggregated costs for providing defined services to NHS patients. NHS providers submit costs annually, which are then used to compute a national cost schedule. The average national costs for these HRGs were collected for the two years: 2019/2020, as well as for a pre-pandemic period (2018/2019).

Alder Hey Children’s Hospital reported the HRG associated with each episode recorded in the baseline and post-intervention data. This captures the number of days the patients spent in critical care units. Patients included in the data displayed eight different Paediatric Critical Care HRGs. Further detail on the characteristics of each of the HRGs included in the analysis is available in Table A1 in the Appendix. Thus, hospital costs for the CDEs were estimated using HRG codes reported by the hospital and the respective National average costs. The expenditure estimation was performed for the baseline group and compared with the post-intervention group.

A comparison between the national average costs and the hospital-reported costs was also performed, using both unadjusted costs and the reference cost index (RCI). This allowed to check the accuracy of cost estimates, as well as to adjust Alder Hey Children’s Hospital costs based on the same case mix delivered at national average cost. Such comparison further contributed to the robustness and reliability of the estimates.

## Results

This section describes the results regarding the comparison of costs between baseline and post-intervention episodes. Comparison between groups was conducted using two different approaches: costs were compared using hospital reported costs, and costs were also compared using national average costs, based on the Healthcare Resource Group associated with each CDE. Table 1 describes the main characteristics of each cohort.

**Table 1:** Key characteristics of each cohort

	Baseline	Post-intervention	Change	Change (%)
Hospital admissions (n)	19,562	19,562	0	0%
Critical Deterioration Events (n)	324	286	-38	-12%
Critical Care Days (n)	3,847	3,457	-390	-10%
Patients (n)	251	225	-26	-10%
Non ICU bed days (n)	86,635	98,363	11,728	14%
Readmissions to critical care within 48 h (n)	48	38	-10	-21%
All cause mortality, whole hospital (n)	64	89	25	39%
Mortality of unplanned admission to critical care (n)	24	32	8	33%

Comparing both cohorts, one can see that for the same overall number of hospital admissions, there was a reduction in the number of Critical Deterioration Events, as well as a reduction on the number of patients experiencing CDEs. In the post-intervention period, however, there was an overall increase in the number of non-ICU bed days and an increase in mortality. These changes are probably linked with the impact of the COVID-19 pandemic during that period.

### Hospital reported costs

In the baseline period, the 324 CDEs had a combined duration of 3,847 critical care days. As discussed before, each CDE in the dataset was capped at 90 days. Nonetheless, such a cap was rarely binding as most CDEs last for less than 90 days. Total cost reported by the hospital for these admissions amounted to £11.8 million, which corresponded to an average daily cost of £3,079.

Comparison between CDEs was made between patients in the baseline and intervention period. These periods were very different in terms of hospital activity, patient population and hospital procedures and processes. Most admissions exposed to the intervention occurred during the Covid-19 pandemic, which increased hospital average costs substantially. Thus, baseline costs were adjusted to account for the additional expenditure that these admissions would face if they had occurred during the Covid-19 pandemic (as the admissions registered in the post-intervention period). The hospital cost accounting department estimated that critical care admission costs increased by 35.4% due to Covid-19. This estimate was done by comparing overall critical care costs before and after Covid-19. Since Covid-19 and the implementation of the DETECT surveillance system happened simultaneously, Covid-19 cost correction is subject to high uncertainty. Nonetheless, adjusting baseline costs for the Covid-19 impact increased total cost to £16 million, which corresponded to an average daily cost of £4,170.

During the intervention period, for the same number of overall admissions, there were 286 CDEs registered, with a combined duration of 3,457 critical care days. Again, these critical care days were capped at 90 days per admission. These implied a total cost above £14 million, which corresponded to an average daily cost of £4,150.

Table 2 displays the comparison of costs for baseline and intervention CDEs, considering both the 90 days cap and the Covid-19 adjustment. For the same overall level of admissions (19,562), there was a reduction from 324 CDE (1.66%) to 286 (1.46%). This corresponds to a reduction of 37 CDEs (-11%). However, such overall reduction is not statistically significant, based on a Fisher exact test (p-value = 0.13).

**Table 2:** Hospital reported costs for Baseline and Intervention CDE (capped at 90 days and with Covid-19 adjustment)

	Baseline	Intervention	Change	Change (%)
Number of events	324.00	286.00	-38.00	-12%
Total days	3,847.00	3,457.00	-390.00	-10%
Days per CDE				
Average	11.87	13.05	1.17	10%
Standard Deviation	15.48	16.18		
Total cost (£)	16,041,992.65	14,347,068.27	-1,694,924.37	-11%
Daily Cost (£)				
Average	4,170.00	4,150.15	-19.85	0%
Standard Deviation	2,460.82	3,221.93		

There was a small increase in the average number of days per CDE, which increased from 11.9 to 13.1 between the baseline and the intervention period. However, this difference was not statistically significant (t-test = 0.19).

Additionally, there was a small decline on the average daily cost adjusted for the Covid-19 impact (-0.5%). However, considering the uncertainty of these estimates, reflected by relatively high standard deviations, the differences in the average daily cost were not significant at the usual significance levels (t-test = 0.44). This suggests that there is no evidence of a significant reduction in the average daily cost, nor in the average number of days per CDE.

These effects combined, resulted in a decrease in expenditure of £1.7 million pounds, which corresponded to an 11% decrease in admission-related expenditures associated with the implementation of DETECT surveillance system. However, as discussed above, such reduction is not statistically significant, considering that the average cost per CDE remained unchanged and that the overall reduction in the number of CDEs was also not significant.

Table A2, available in the Appendix, provides the same analysis without the Covid-19 adjustment. Without Covid-19 adjustment, overall costs would increase relative to the baseline. Considering the high degree of uncertainty associated with the Covid-19 adjustment, hospital reported data suggests that there is no statistical evidence that major savings were achieved following the implementation of the DETECT surveillance system.

### Healthcare resource groups costs

Alder Hey Children’s Hospital reported the HRG associated with each episode in the baseline and intervention data. Based on these HRGs and on the average unit cost published by the NHS (see Table A3 available in the Appendix), we estimated Alder Hey Children’s Hospital expenditure. Patients included in the study displayed eight different Paediatric Critical Care HRGs.

Table 3 displays the comparison of the HRG units (which reflects the total number of admissions days) recorded between baseline and intervention patients. Overall, when comparing both periods, we observed a sizeable reduction in the volume of most HRGs. The only three HRGs with more activity (bed-days) in the post-intervention period relative to the baseline were Enhanced Care and Basic Critical Care (XB09Z and XB07Z), as well as Advanced Critical Care 5 (XB01Z). Within the Paediatric Critical Care HRGs, Enhanced Care and Basic Critical Care are the least complex HRGs and the two with the lowest costs. As each unit corresponds to one bed-day, we observed that the total number of critical care days decreased from almost 3800 in the baseline to approximately 3300 in the post-intervention data. This represents a decrease of 455 critical care days for the matched cohort of 19,562 admissions.

**Table 3:** Paediatric Critical Care HRG comparison between baseline and intervention admission

Code^a^	HRG	Baseline	Intervention	Change	Change (%)
XB01Z	Advanced Critical Care 5	68	76	8	12%
XB02Z	Advanced Critical Care 4	56	17	-39	-70%
XB03Z	Advanced Critical Care 3	228	181	-47	-21%
XB04Z	Advanced Critical Care 2	633	587	-46	-7%
XB05Z	Advanced Critical Care 1	918	766	-152	-17%
XB06Z	Intermediate Critical Care	1 554	1 292	-262	-17%
XB07Z	Basic Critical Care	183	234	51	28%
XB09Z	Enhanced Care	152	184	32	21%
	Total	3 792	3 337	-455	-12%

^a^HRG codes are ordered from the most complex critical care admissions (XB01Z) to the least complex one (XB09Z)

The overall reduction in HRG codes is mostly related with the overall decline in the number of CDEs between the baseline and intervention period (324 and 286, respectively). However, as mentioned above, the overall reduction in the number of CDEs was not statistically significant.

The reduction in the number of days in critical care following a CDE has a direct implication in terms of costs. We used 2019/2020 NHS costs to estimate the potential saving by comparing the overall cost associated with these CDE between patients in the baseline and in the post-intervention period. As expected, we observed a reduction in expenditures for most HRG codes, as described by Table 4. Overall, there was a reduction close to £1.1 million when comparing the costs associated with the CDEs between baseline and post-intervention patients. This represents a reduction of 13% relative to the baseline cost.

**Table 4:** Estimated HRG Costs for patients in the Baseline and Post-Intervention period (£)

Code^a^	HRG	National average unit cost (£)	Baseline (£)	Intervention (£)	Change (£)	Change (%)
XB01Z	Advanced Critical Care 5	4 491.84	305 444.84	341 379.52	35 934.69	12%
XB02Z	Advanced Critical Care 4	3 808.28	213 263.41	64 740.68	-148 522.73	-70%
XB03Z	Advanced Critical Care 3	2 844.61	648 571.91	514 875.07	-133 696.84	-21%
XB04Z	Advanced Critical Care 2	2 673.76	1 692 490.55	1 569 497.55	-122 992.99	-7%
XB05Z	Advanced Critical Care 1	2 224.96	2 042 515.56	1 704 321.27	-338 194.30	-17%
XB06Z	Intermediate Critical Care	1 867.66	2 902 347.85	2 413 020.22	-489 327.63	-17%
XB07Z	Basic Critical Care	1 572.52	287 771.33	367 969.90	80 198.57	28%
XB09Z	Enhanced Care	1 023.38	155 554.22	188 302.48	32 748.26	21%
	Total		8 247 959.66	7 164 106.68	-1 083 852.98	-13%

^a^HRG codes are ordered from the most complex critical care admissions (XB01Z) to the least complex one (XB09Z)

This effect is mostly explained by the non-significant reduction in the number of CDEs when comparing both periods. The reduction in the number of CDEs, contributed to reduce the number of critical care days, leading to a cost reduction. Considering that the reduction in the number of CDEs was not significant, there is also uncertainty regarding the cost savings estimated.

Despite the overall non-significant reduction in the overall number of CDE, one can also investigate the composition of each CDE: by analysing the number and type of codes per CDE, as described by Table 5.

**Table 5:** Estimated HRG Costs for patients in the Baseline and Post-Intervention period per CDE

Code^a^	HRG	Baseline		Intervention		Change	
		Mean	Standard Deviation	Mean	Standard Deviation	Mean	t-test (*p*-value)
XB01Z	Advanced Critical Care 5	0.24	2.02	0.32	1.93	0.09	0.32
XB02Z	Advanced Critical Care 4	0.18	1.11	0.06	0.34	-0.12	0.05
XB03Z	Advanced Critical Care 3	0.64	2.12	0.69	2.46	0.06	0.40
XB04Z	Advanced Critical Care 2	1.52	4.80	1.71	4.53	0.18	0.34
XB05Z	Advanced Critical Care 1	2.26	4.92	2.15	4.32	-0.11	0.40
XB06Z	Intermediate Critical Care	4.21	7.60	4.26	7.73	0.05	0.47
XB07Z	Basic Critical Care	0.57	7.60	0.80	7.73	0.24	0.04
XB09Z	Enhanced Care	0.54	1.08	0.69	1.74	0.15	0.18
	Total	10.16	12.13	10.69	12.37	0.53	0.32

^a^HRG codes are ordered from the most complex critical care admissions (XB01Z) to the least complex one (XB09Z)

Overall, the average number of codes per CDE increased from 10,16 to 12,37, although such increase was not significant as well. This suggests that the average number of days per CDE remained did not change considerably – similarly to what was found when using hospital-reported costs.

In terms of composition of each CDE, one can observe a significant reduction in the number of days in an advanced critical care code (XB02Z) and an increase in the number of days in an intermediate critical care code (XB06Z). This suggests some potential de-escalation of care for these admissions.

Since the average number of codes per CDE remained relatively stable, without major shifts between different codes, the estimated cost per CDE did not decrease as well. Thus, all cost reductions were driven by a non-significant reduction in the overall number of CDEs, and not by a reduction in the unit cost of each CDE, as described by Table 6.

**Table 6:** Estimated HRG Costs per CDE (£)

Code^a^	HRG	Baseline	Intervention	Change	Change (%)
XB01Z	Advanced Critical Care 5	1 064.80	1 457.35	392.55	37%
XB02Z	Advanced Critical Care 4	690.34	2 39.94	-450.40	-65%
XB03Z	Advanced Critical Care 3	1 808.58	1 965.19	156.61	9%
XB04Z	Advanced Critical Care 2	4 074.56	4 562.03	487.47	12%
XB05Z	Advanced Critical Care 1	5 031.28	4 791.67	-239.61	-5%
XB06Z	Intermediate Critical Care	7 868.74	7 963.12	94.38	1%
XB07Z	Basic Critical Care	889.11	1 259.75	370.64	42%
XB09Z	Enhanced Care	551.78	704.43	152.65	28%
	Total	2 175.09	2 146.87	-28.22	-1%

^a^HRG codes are ordered from the most complex critical care admissions (XB01Z) to the least complex one (XB09Z)

Using the same cost schedule to analyse both the baseline and intervention data prevents potential cost bias arising from the Covid-19 pandemic. Thus, no adjustment to these costs is required, contrary to the approach followed in the hospital reported cost section. Overall, savings were aligned between both approaches. We estimated that implementation of the DETECT surveillance system reduced critical care admissions costs by 11% and 13%, depending on whether we look at Hospital Reported Costs or Healthcare Resource Groups, respectively. A sensitivity analysis was also performed using national average costs from 2018/2019, to capture pre-pandemic trends. Estimates did not change significantly.

Nonetheless, there is significant asymmetry in hospital costs across the country. The NHS has developed the reference cost index (RCI) which measures the relative cost difference between NHS providers. This index shows the actual cost of a provider’s case mix compared with the same case mix delivered at national average cost. Table A4, available in the Appendix, displays the Reference Cost Index for Alder Hey Children’s Hospital. Using the most recent year available with Market Forces (MFF) adjustment (2018) it shows that Alder Hey’s costs are typically slightly above the national average. Regarding critical care services, Alder Hey Children’s Hospital costs were, on average, 18% higher than national average. Among other factors, this may reflect variation in the admission thresholds to PICU, nationally. In fact, since some PICUs are co-located with HDUs, the threshold for admission in these units might be lower.

The overall estimated costs are lower than the ones reported by Alder Hey Children’s Hospital cost accounting. In the baseline period, hospital reported costs amount to £16.0 million, considering the Covid-19 adjustment, while HRG expenditure amounts to £8.2 million. Similarly, in the intervention period, hospital reported costs are £14.3 million compared with a total cost of £7.2 million with HRG costs.

The difference can be explained by cost differences between the hospital and the national average (used to compute unit costs), as well as methodological differences regarding the type and scope of the costs included. According to the Reference Cost Index, Alder Hey’s critical care costs are typically 18% above the paediatric national average for critical care. This reflects potential differences in terms of the case-mix and differentiation of each centre. Therefore, extrapolation of costs implies an adjustment of Alder Hey’s costs relative to average. This adjustment decreases overall hospital reported daily costs.

Nonetheless, hospital reported costs after adjustment remained higher than the national average costs for paediatric critical care in 2019/2020. HRG cost were 49% and 50% lower than hospital costs for the baseline and intervention period respectively. This gap decreased to 39% and 41% after the adjustment. However, the scope of the paper is focused on the cost change – and not on the level of costs – which is not affected by this (savings after adjustments remain at 11% and 13%, depending on whether one uses hospital reported costs or HRG costs, respectively).

## Discussion

The DETECT surveillance system aims to proactively screen paediatric patients for early signs of serious deterioration or sepsis, thereby reducing complications and emergency transfers to critical care following deterioration in hospital. Avoiding the unexpected escalation of care for these patients can contribute to cost savings through an overall reduction in the number of CDEs registered, as well as through a reduction on the average cost per CDE. Our results suggest a decrease in costs following the implementation of the DETECT surveillance system, there is an annual reduction in costs of between £1.1 and 1.7 M. Although the reduction is not statistically significant in our analysis, it is a consistent finding using both the hospital costs and the HRG costs, and could be an intervention that could support efficiency saving in the NHS.

In this paper we estimated the cost change associated with the intervention. We employed two complementary approaches; hospital reported costs for patients who experienced CDEs in the baseline and the post-intervention period; and data on NHS average costs, based on HRG codes for patients.

On average, hospital reported costs were higher than the respective HRG national average costs used by the NHS. The difference is explained by three main effects. Firstly, HRG data reflect average national costs, which differ from each specific hospital cost. We accounted for this difference by using the Reference Cost Index provided by the NHS, which corrects for average cost differences between hospitals. Correcting for the permanent cost difference, the gap on reported costs decreases from 49 to 39% for baseline patients, and from 50 to 41% for patients exposed to the intervention.

Secondly, there are methodological differences regarding the scope of the costs reported. For instance, the mandatory reporting of costs from hospitals to the NHS uses a top-down approach [15], while hospital reported costs can have additional detail, as discussed above.

Thirdly, HRG costs are based on all elective and emergency admissions to critical care. It is clear from these data that emergency transfer to critical care following in-patient deterioration incurs significantly higher costs patient-level costs per episode compared to aggregated HRG costs. The mismatch in HRG pay-back for in-patient deterioration episodes highlights that early intervention and stabilisation of in-patient deterioration could yield benefits in minimising excess expenditure for patient care delivery, which is an important point for hospitals seeking to deliver cost-effective care.

Our results suggest a decrease in costs following the implementation of the DETECT surveillance system. However, such reduction is not statistically significant, as it is driven by an overall non-significant reduction in the number of CDE: when comparing both periods, we observed a reduction in the volume of most HRGs (the total number of critical care days decreased from 3,800 to approximately 3300 days in the post-intervention data), reflecting a reduction in the number of CDEs, which decreased from 324 in the baseline period to 286 in the post-intervention period. Using the 2019/2020 NHS costs, we estimated a reduction in expenditures for most HRG codes.

Overall, there was a reduction of 13% relative to the cost for baseline patients. When looking to hospital reported costs, we estimated a reduction of 11%. These savings were not statistically significant. Further research is required to enlarge the sample size and to collect data after the impact of Covid-19 to provide a more accurate comparison. Nonetheless, even though our sample size did not allow for the identification a statistically significant effects, there is suggestive evidence that this intervention may contribute to reduce costs.

As discussed above, such savings can be attributed to either a decline in the number of CDEs or to a reduction in the average cost per CDE. The latter would happen if the CDEs for the post-intervention data were, on average, less severe than for baseline patients. Our results suggest that all savings are explained by the overall reduction in the number of CDEs. This reduction is, however, not statistically significant. Moreover, the estimated cost per CDE does not seem to decrease – suggesting that each CDE implies a similar cost. Contrary to previous studies [14] we did not find a substantial average cost reduction associated with each episode. Still, we find suggestive evidence that there is a reduction in the number of days in an advanced critical care code and an increase in the number of days in an intermediate critical care core. This may reflect lower chances of death and potentially lower costs. Given the low number of observations in our sample, these changes were not enough to be translated into statistically significant cost reductions.

These cost estimates can be seen as a proxy for the opportunity cost of those critical care beds. If the intervention allows for the reduction of CDEs, and for the reduction in the number of critical care days, then these cost savings represent the value of the resources that will be available to be used in other patients and purposes. However, our results do not provide enough evidence to support this hypothesis: even though a cost reduction was estimated in our sample, such reduction was not statistically significant.

Some considerations must also be discussed regarding the external validity of these results. As the intervention took place in a single hospital, extrapolations of these results to other hospitals must be carefully made. It is likely that hospital-specific characteristics (such as patient case mix, pathways, and processes for recognising and responding to deterioration and for admission to critical care) may have some impact on the estimates.

One major concern is the fact that the two groups of patients were not observed simultaneously. In particular, the post-intervention data was exposed to the Covid-19 pandemic, which could have affected estimates. Nonetheless, it was possible to collect data on a matched cohort which allowed this concern to be minimised. Further research will be required to fully control for this limitation and overcome potential sample size issues.

Unit costs were also adjusted to capture average cost increases relative to the Covid-19 pandemic. However, adjustments to Covid-19 related cost increases were subject to high uncertainty. This adjustment was estimated by comparing overall critical care costs before and after Covid. To some extent, costs post-Covid may also include the effect of the DETECT surveillance system. Nonetheless, the estimate of a 35,4% cost increase in critical care due to Covid seems relatively conservative. In fact, overall health care budget in the UK increased by roughly 25% due to Covid^2^. It is likely that critical care costs increased above the average, with some European studies estimating critical care costs increases due to Covid above 70% [16].

Nonetheless, to deal with the uncertainty related with the Covid-19 cost adjustment, the hospital reported costs perspective was complemented with an analysis of national level costs, which improves the reliability of the estimates. Variations are expected to occur depending on how each hospital performs relative to the national average.

## Conclusion

This study provides a discussion on the potential direct critical care cost savings arising from the implementation of the DETECT surveillance system. Although the overall reduction of the number of CDEs is not statistically significant, the potential saving of between £1–1.7 million /year (or ~ 10% hospital costs due to CDE) is one that would be welcomed by hospitals seeking to make efficiency savings.

We compared the cost associated with CDEs experienced by 19,562 hospital admissions in the baseline period and the same number of admissions during the DETECT surveillance system period, by combining hospital reported costs with NHS average costs, into an integrated perspective on how costs are affected by the DETECT surveillance system. We observed a reduction in the total number of critical care days leading to a cost reduction of between £1.1 and £1.7 million/year. This implies an 11%—13% saving relative to the baseline cost, depending on the approach followed (hospital reported costs versus NHS average costs). These cost reductions were driven by an overall non-significant reduction in CDEs and not by a reduction in the unit cost of each CDE.

These estimates focus exclusively on critical care costs, both for hospital reported and HRG costs. Total ward costs and changes associated with potentially different lengths of stay were not included in the scope of this paper. Moreover, these estimates reflect the direct cost change associated with CDEs but do not include the cost of acquiring and implementing the technology. Even if not translated directly in terms of cost savings, early warning systems may contribute to improve the quality of care and enhance patient outcomes.

Overall, our results support the hypothesis that reducing CDEs has a direct impact on critical care costs and highlights the importance of surveillance technologies in anticipating patients’ deterioration and avoiding care escalation.

## Supplementary Information

**Additional file 1: Table A1.** Description of HRG codes. **Table A2.** Hospital reported costs for Baseline and Intervention CDE (capped at 90 days and without Covid-19 adjustment). **Table A3.** National Schedule of NHS Costs (2019-20) - All NHS trusts and NHS foundation trusts. **Table A4.** Reference Cost Index (100 = national average).

## Data Availability

The datasets generated and/or analysed during the current study are not publicly available due to privacy concerns and health-related individual data, but are available from the corresponding author on reasonable request.

## Abbreviations

CDE: Critical Deterioration Event
DETECT: Dynamic Electronic Tracking and Escalation to reduce critical Care Transfers
LSOA: Lower Super Output Area code
MFF: Market forces adjustment
NHS: National Health Service
PPI: Patient and Public involvement

## Footnotes

1 Further details on the NHS Costing Methodology available at https://www.england.nhs.uk/costing-in-the-nhs/approved-costing-guidance/

2 https://www.bma.org.uk/advice-and-support/nhs-delivery-and-workforce/funding/health-funding-data-analysis
