# Supplementary material for: Using technology to reduce critical deterioration (the DETECT study): a cost analysis of care costs at a tertiary children's hospital in the United Kingdom
Source: BMC Health Serv Res. 2023 Jul 4;23:725. doi: 10.1186/s12913-023-09739-3 (PMC10318736; doi:10.1186/s12913-023-09739-3)
Supplement: Supplementary file 1 — Additional file 1: Table A1. Description of HRG codes. Table A2. Hospital reported costs for Baseline and Intervention CDE (capped at 90 days and without Covid-19 adjustment). Table A3. National Schedule of NHS Costs (2019-20) - All NHS trusts and NHS foundation trusts. Table A4. Reference Cost Index (100 = national average). [file 12913_2023_9739_MOESM1_ESM.docx]

# **Appendix**

***Table A1:*** *Description of HRG codes*

| **Code** | **Label** | **Description** |
| --- | --- | --- |
| XB01Z | Paediatric Critical Care, Advanced Critical Care 5 | Extracorporeal membrane oxygenation (ECMO) |
|  |  | Extracorporeal Life Support (ECLS) including Ventricular Assist Device (VAD) |
|  |  | Aortic balloon pump |
| XB02Z | Paediatric Critical Care, Advanced Critical Care 4 | Invasive Mechanical Ventilation or |
|  |  | Advanced Respiratory Support (Jet Ventilation or HFOV) and at least one of: |
|  |  | Burns >79% BSA |
|  |  | >80 ml/kg volume boluses |
| XB03Z | Paediatric Critical Care, Advanced Critical Care 3 | Invasive Mechanical Ventilation or |
|  |  | Advanced Respiratory Support (Jet Ventilation or HFOV) and at least one of: |
|  |  | Haemofiltration |
|  |  | Haemodialysis |
|  |  | Peritoneal dialysis |
|  |  | Burns 50-79% BSA |
|  |  | Extracorporeal liver Support (MARS) |
|  |  | Exchange transfusion |
|  |  | iNO |
|  |  | Surfactant |
|  |  | Plasmafiltration |
| XB04Z | Paediatric Critical Care, Advanced Critical Care 2 | Advanced Respiratory Support (ARS) or |
|  |  | Frequency Oscillatory Ventilation (HFOV) or |
|  |  | Invasive Mechanical Ventilation and at least one of: |
|  |  | Vasoactive infusion |
|  |  | ICP monitoring |
|  |  | Burns 20-49% BSA |
|  |  | Intravenous thrombolysis |
|  |  | CPR in last 24 hrs |
| XB05Z | Paediatric Critical Care, Advanced Critical Care 1 | Invasive Mechanical Ventilation (IMV) |
|  |  | Non-invasive ventilation / CPAP |
|  |  | PLUS one or more of: |
|  |  | Vasoactive infusion CPR in last 24 hrs |
|  |  | >80 ml/kg volume boluses Intravenous thrombolysis |
|  |  | Haemofiltration Burns >20% BSA |
|  |  | Haemodialysis iNO / Surfactant |
|  |  | Peritoneal dialysis Exchange transfusion |
|  |  | Plasmafiltration ICP monitoring |
|  |  | Extracorporeal liver Support (MARS) |
| XB06Z | Paediatric Critical Care, Intermediate Critical Care | Airway: Nasopharyngeal airway |
|  |  | Airway: Care of tracheostomy (first seven days of episode only) |
|  |  | Breathing: Non-invasive ventilation (including CPAP and BiPAP) |
|  |  | Breathing: Long-term ventilation via a tracheostomy |
|  |  | Circulation: >80 ml/kg volume boluses |
|  |  | Circulation: Vasoactive infusion (including inotropes and prostaglandin) |
|  |  | Circulation: Temporary external pacing |
|  |  | Circulation: Cardiopulmonary resuscitation in the last 24 hours |
|  |  | Diagnosis: Acute renal failure requiring dialysis or haemofiltration |
|  |  | Diagnosis: Status epilepticus requiring treatment with continuous IV infusion |
|  |  | Monitoring: |
|  |  | Monitoring: Invasive arterial monitoring |
|  |  | Monitoring: Central venous pressure monitoring |
|  |  | Monitoring: Intracranial monitoring / external ventricular drain |
|  |  | Other: Exchange transfusion |
|  |  | Other: Intravenous thrombolysis |
|  |  | Other: Extracorporeal liver support (MARS) |
|  |  | Other: Plasmafiltration |
|  |  | Other: Epidural infusion |
| XB07Z | Paediatric Critical Care, Basic Critical Care | Airway: Upper airway obstruction requiring nebulised adrenaline |
|  |  | Breathing: Apnoea – recurrent |
|  |  | Breathing: Oxygen therapy plus continuous pulse oximetry plus ECG monitoring |
|  |  | Breathing: Nasal high flow therapy |
|  |  | Circulation: Arrhythmia requiring IV anti-arrhythmic therapy |
|  |  | Diagnosis: Severe asthma (IV bronchodilator / continuous nebulisers) |
|  |  | Diagnosis: Diabetic ketoacidosis requiring continuous insulin infusion |
|  |  | Other: Reduced level of consciousness (GCS <=12) and hourly GCS monitoring |
| XB09Z | Paediatric Critical Care, Enhanced Care | Enhanced observation and monitoring without any added organ support. |

***Table A2:*** *Hospital reported costs for Baseline and Intervention CDE (capped at 90 days and without Covid-19 adjustment)*

|  | **Baseline** | **Intervention** | **Change** |
| --- | --- | --- | --- |
| Number of events | 324 | 286 | -38 |
| Total days | 3 847 | 3 457 | -390 |
| Average daily cost (£) | 3 078.99 | 4 150.15 | 1 071.16 |
| Total cost (£) | 11 840 888.80 | 14 347 068.27 | 2 506 179.47 |

***Table A3:*** *National Schedule of NHS Costs (2019-20) - All NHS trusts and NHS foundation trusts*

| **HRG** | **Activity** | **Unit Cost (£)** | **Total Cost (£)** |
| --- | --- | --- | --- |
| XB01Z | 1 988 | 7 646.26 | 15 200 769.96 |
| XB02Z | 1 282 | 4 903.62 | 6 286 440.80 |
| XB03Z | 4 297 | 3 301.36 | 14 185 949.53 |
| XB04Z | 15 379 | 2 873.50 | 44 191 529.11 |
| XB05Z | 32 736 | 2 493.31 | 81 621 102.55 |
| XB06Z | 55 480 | 1 581.90 | 87 763 621.19 |
| XB07Z | 31 747 | 1 478.16 | 46 927 117.93 |
| XB09Z | 32 257 | 907.43 | 29 270 933.71 |

***Table A4:*** *Reference Cost Index (100 = national average)*

| **Index** | **2016** | **2017** | **2018** | **2019** | **D.17-16** | **D.18-17** | **D.19-18** |
| --- | --- | --- | --- | --- | --- | --- | --- |
| *MFF Adjusted* |  |  |  |  |  |  |  |
| Organisation-Wide Index | 111 | 103 | 107 |  | -8% | 4% |  |
| Critical Care Services | 115 | 90 | **118** |  | -22% | 32% |  |
| Unbundled | 110 | 120 | 116 |  | 9% | -3% |  |
| *MFF Unadjusted* |  |  |  |  |  |  |  |
| Org-Wide Index | 105 | 97 | 101 | 97 | -8% | 3% | -4% |
| Critical Care Services | 107 | 84 | 110 | 109 | -21% | 31% | -1% |
| Unbundled | 106 | 115 | 112 | 113 | 8% | -2% | 1% |
